# Visual marker-based augmented reality for intraoperative ultrasound in minimally invasive surgery

**DOI:** 10.1007/s00464-025-12326-8

**Published:** 2025-10-23

**Authors:** Takeshi Takamoto, Yusuke Kazami, Yujiro Nishioka, Akihiko Ichida, Nobuhisa Akamatsu, Yoshikuni Kawaguchi, Kiyoshi Hasegawa

**Affiliations:** Hepato-Biliary-Pancreatic Surgery Division, Artificial Organ and Transplantation Division, Department of Surgery, Graduate School of Medicine, The University of Tokyo, Tokyo, Japan

**Keywords:** Augmented reality, Ntraoperative ultrasonography, Minimally invasive surgery, Visual marker tracking, Eye tracking

## Abstract

**Background:**

Intraoperative ultrasonography (IOUS) is essential in minimally invasive hepatobiliary–pancreatic surgery but requires surgeons to alternate attention between laparoscopic and ultrasound monitors, disrupting workflow. We developed Flag AR, an augmented reality system that superimposes real-time ultrasound images onto laparoscopic displays using visual marker tracking.

**Methods:**

This prospective observational study included 14 patients undergoing minimally invasive hepatobiliary–pancreatic procedures between March and April 2025. Three experienced surgeons performed target identification tasks using both Flag AR and conventional IOUS. Eye-tracking analysis measured gaze shifts and visual focus time using wearable eye-tracking glasses.

**Results:**

Twenty-six anatomical targets were evaluated, including liver tumors (*n* = 13), gallbladder tumors (*n* = 4), and vascular structures (*n* = 9). Although task completion time showed no significant difference between modalities (23.8 vs. 22.1 s, *P* = 0.351), Flag AR significantly reduced gaze shifts compared to conventional IOUS (1.8 vs. 8.4 shifts, *P* < 0.001) and increased time focused on a single monitor (97.9% vs. 73.8%, *P* < 0.001).

**Conclusion:**

Flag AR represents the first clinical application of purely visual marker-based ultrasound overlay technology in minimally invasive surgery. By enabling sustained visual focus on a single display, this system improves surgeon workflow efficiency during IOUS procedures without compromising task performance, potentially enhancing spatial visualization and reducing cognitive workload in complex surgical procedures.

**Supplementary Information:**

The online version contains supplementary material available at 10.1007/s00464-025-12326-8.

The resection of parenchymal organs such as the liver and pancreas presents technical challenges, primarily due to the inability to directly visualize internal anatomical structures. To address this limitation, various surgical navigation systems, such as intraoperative cholangiography, indocyanine green fluorescence imaging [1, 2], and optical or electromagnetic tracking [3], have been developed to supplement the operative field with essential anatomical information. These modalities assist surgeons in identifying tumors and vascular structures embedded within the organ parenchyma, thereby enhancing surgical precision and safety. With the recent increasing adoption of minimally invasive surgery (MIS) [4–6], the demand for effective intraoperative navigation tools has intensified. MIS restricts surgeons to unidirectional visual information on a shared monitor and eliminates the multifaceted visual and tactile feedback available in open procedures.

Among the available modalities, intraoperative ultrasonography (IOUS) has been used as a real-time, high-resolution imaging tool and has become indispensable in liver and pancreatic surgery since its introduction in the 1980s [7, 8]. IOUS facilitates accurate tumor localization and delineation of major vascular structures, contributing to intraoperative decision-making and enhancing surgical accuracy. In the context of where surgeons must rely exclusively on visual information, the value of IOUS is even more pronounced. However, the application of IOUS in MIS introduces unique challenges [9, 10]. Surgeons are forced to alternate their attention between the laparoscopic monitor and a separate ultrasound display, disrupting workflow and situational awareness [11, 12]. Moreover, miniaturized IOUS probes designed for MIS require distinct handling and often suffer from unstable contact and limited scanning angles, compromising the reliability and completeness of intraoperative imaging.

To overcome these technical limitations, we have developed “Flag AR,” an IOUS system incorporating advanced augmented reality (AR) technology that superimposes isometric real-time ultrasonographic images directly onto the endoscopic visualization platform. In this study, we evaluated Flag AR in clinical practice during minimally invasive hepatobiliary surgery, with particular interest in assessing whether this system improves IOUS usability.

## Methods

### Study design and participants

This was a prospective observational study in a single institution. Between March and April 2025, patients scheduled to undergo liver resection, cholecystectomy, pancreatic resection, or diagnostic laparoscopy using either laparoscopic- or robot-assisted procedures were enrolled. Written informed consent was obtained from all participants prior to enrollment. The study protocol was approved by the Institutional Review Board of our hospital (IRB No. 2024473NIe).

### Flag AR

Flag AR is an IOUS system developed in conjunction with a redesigned laparoscopic linear ultrasound probe (L43LAP; FUJIFILM Corporation, Tokyo, Japan). This system enables real-time superimposition of ultrasound images onto laparoscopic video feeds, thereby facilitating intuitive intraoperative spatial orientation. The L43LAP is the successor to the conventional articulating laparoscopic linear probe (L44LA; FUJIFILM Corporation, Tokyo, Japan), featuring a slender, rod-shaped design with an articulating tip, specifically optimized for minimally invasive procedures.

**Fig. 1:**
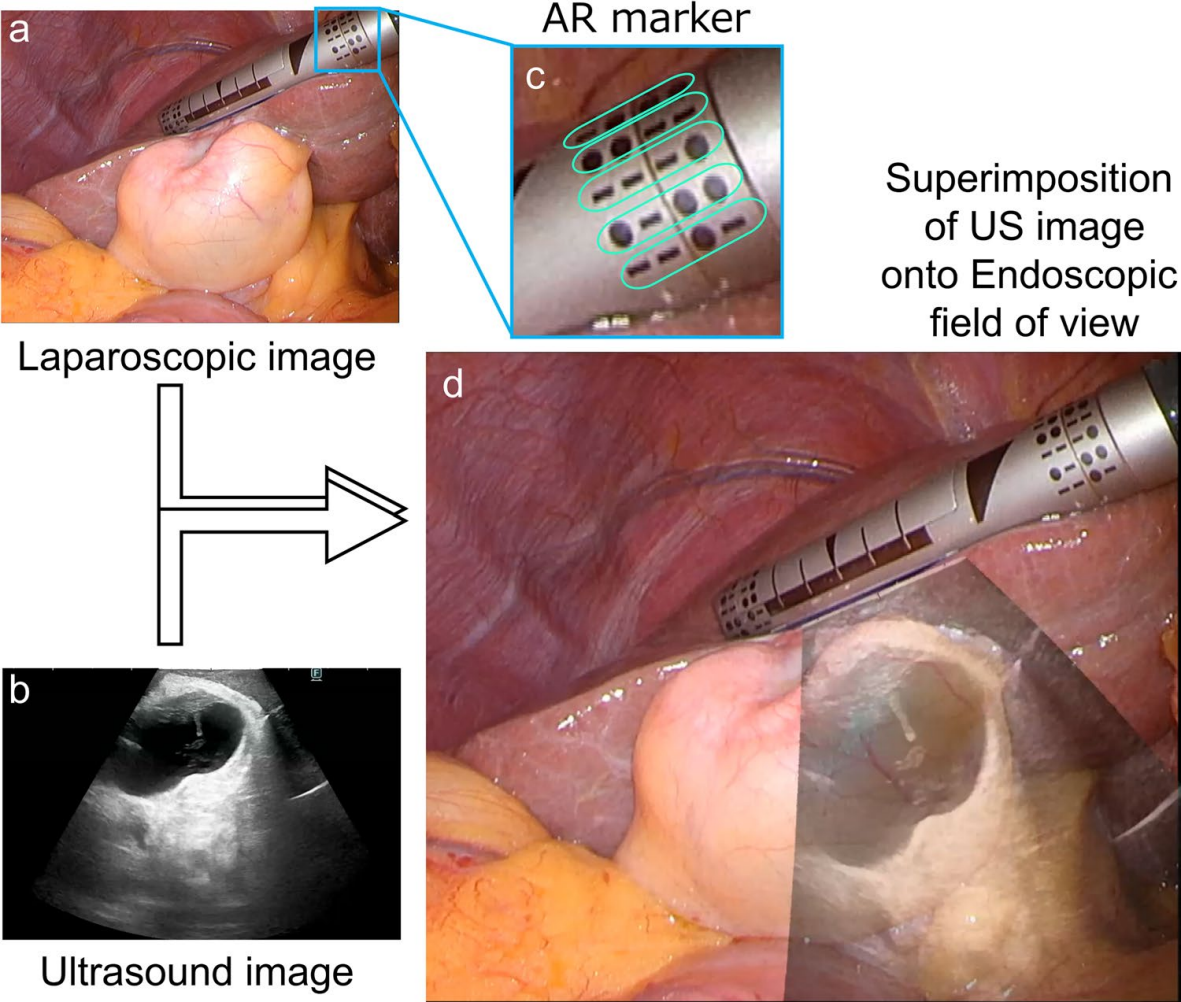
Overview of the Flag AR. **a** Laparoscopic view showing the L43LAP ultrasound probe positioned on liver surface to examine gallbladder tumor during minimally invasive surgery. **b** Corresponding ultrasound image investigating inside of gallbladder and gall bladder tumor. **c** Magnified view of the AR marker system on the probe tip, showing 13 distinct pattern rows arranged circumferentially around the probe shaft. Each pattern row consists of four symbols with unique sequence configurations. **d** Real-time ultrasound image superimposition onto the laparoscopic field of view using Flag AR technology, with the ultrasound image overlaid at 1:1 scale

The L43LAP probe tip is equipped with AR tracking technology that uses multiple small visual markers integrated into the tip. The AR marker system consists of 13 distinct pattern rows arranged circumferentially around the probe shaft. Each pattern row comprises four symbols (solid square, solid circles, or dashes) aligned along the longitudinal axis, with each row possessing a unique sequence configuration. This uniqueness enables precise identification of the probe position corresponding to the symbols recognized within the laparoscopic image (Fig. 1). The AR visualization of ultrasound data is achieved through perspective projection transformation, utilizing correspondence points between image coordinates and world coordinates to establish accurate spatial relationships. Flag AR processes the laparoscopic video stream to identify these AR markers and calculates the probe’s tip location and direction with high precision in real time. Based on this information, the corresponding ultrasound image is accurately superimposed onto the laparoscopic field of view. This superimposition appears as if the ultrasound image were projected like a flag attached to the shaft of the probe—where the probe serves as the flagpole and the ultrasound image is displayed as the flag itself. The overlaid ultrasound image is rendered at a 1:1 scale with the laparoscopic view, allowing for precise visual integration and spatial correlation between the two modalities.

### Technology implementation and adoption

The Flag AR system was designed for seamless integration into existing laparoscopic setups with minimal additional equipment requirements. The system consists of a standard ultrasound unit, the modified laparoscopic probe, and a software module that processes the laparoscopic video feed in real time. Implementation required a brief 3-min setup procedure and a 15-min training session for surgical staff. The learning curve for experienced laparoscopic surgeons was minimal, with proficiency achieved within 1–2 procedures. The system demonstrated high reliability, with marker detection accuracy exceeding 95% under standard laparoscopic lighting conditions.

### Targets identification procedure

Ultrasound procedures using Flag AR were conducted by three experienced surgeons who had completed standard surgical hand scrubbing and entered the sterile field. All participating surgeons were certified expert surgeons of the Japanese Society of Hepato-Biliary-Pancreatic Surgery with over 15 years of surgical experience and more than 100 cases of minimally invasive hepatobiliary-pancreatic surgery. The surgeons manipulated the ultrasound probe to locate anatomical targets—such as hepatic tumors, gallbladder masses, or vascular structures—that had been identified on preoperative CT images.

Each session was conducted under two conditions: with and without the Flag AR. To minimize potential allocation bias, the order of the two conditions—Flag AR first or conventional IOUS first—was alternated sequentially for each target. For each session, we measured the time required to identify the target structures and the total gaze trajectory distance using an eye-tracking device for subsequent comparative analysis.

### Gaze tracking analysis using eye-tracking glasses

**Fig. 2:**
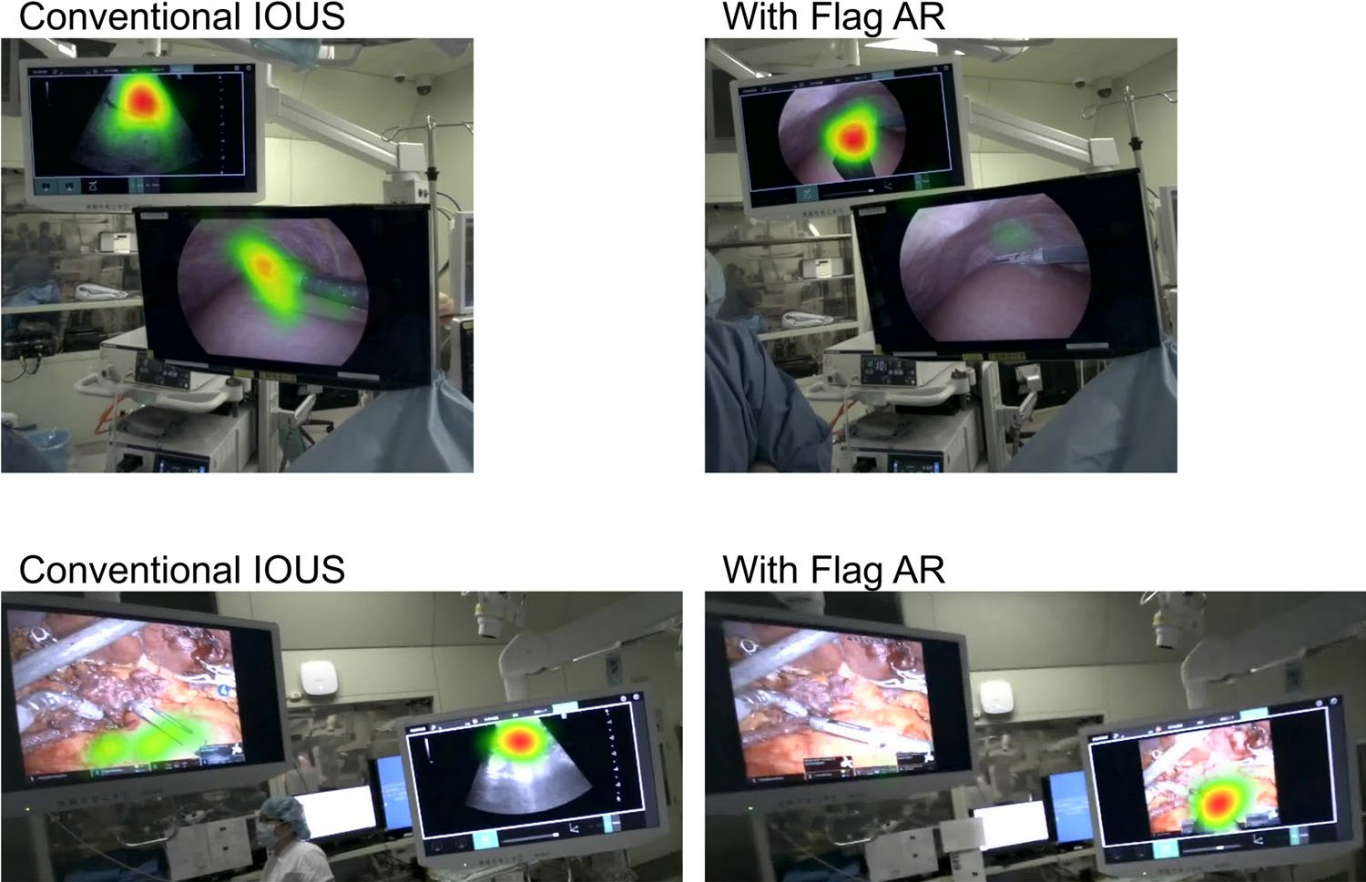
Surgeon gaze pattern analysis during target identification. Left panels (Conventional IOUS): Surgeons are required to monitor both the endoscopic display (lower monitor) and separate ultrasound monitor (upper monitor), resulting in dispersed visual attention across multiple displays as shown by the heat map overlay. Right panels (With Flag AR): The AR superimposition system enables surgeons to focus visual attention predominantly on the endoscopic monitor, as the ultrasound image is directly overlaid onto the laparoscopic field of view. Heat map visualization clearly demonstrates concentrated gaze patterns on a single display when using Flag AR, corresponding to the quantitative finding of 97.9% single-monitor focus time compared to 73.8% with conventional IOUS

A wearable eye-tracking system designed for real-world research applications (Tobii Pro Glasses 3; Tobii Technology, Danderyd, Sweden) was used to monitor eye movements during intraoperative procedures. The device is equipped with four eye cameras and 16 near-infrared illuminators embedded in anti-reflective, scratch-resistant lenses, allowing for precise binocular tracking at a sampling rate of 50 or 100 Hz. It employs a 3D eye model to ensure accurate gaze point calculation, even during head movements. A full HD scene camera with a 106° field of view captures the participant’s environment, thereby enabling comprehensive analysis of gaze behavior. Calibration was conducted prior to the target searching session using a one-point manual procedure with a calibration card to ensure optimal accuracy. The lightweight design (head unit: 76.5 g) permits natural head and body movement, minimizing interference with the scrubbed-in surgeon’s motion during ultrasound manipulation and target localization in the sterile field. All scene videos and gaze trajectories were recorded and subsequently analyzed postoperatively (Fig. 2).

### Statistics

Data are expressed as a median and range. Since each surgeon performed both conditions (with and without Flag AR), differences in target identification time and total gaze trajectory distance were analyzed using paired *t* tests for normally distributed continuous variables and Wilcoxon signed-rank test for non-normally distributed continuous variables. Continuous data were summarized as median and range, and were compared using paired t-tests or Wilcoxon signed-rank tests as appropriate based on data distribution. All statistical tests were performed using statistical software (JMP, version 17; SAS Institute, Cary, NC, USA). A *P* value of < 0.05 was considered statistically significant.

## Results

### Patient characteristics

Fifteen patients were initially enrolled for Flag AR evaluation. One patient was excluded due to the inability to reach the preoperatively planned target (S7 tumor) with the ultrasound probe, making measurement impossible. Therefore, 14 patients were included in the final analysis with a median age of 68 years (range: 41–92 years). Seven patients were female (50%). Eleven procedures were laparoscopic (79%) and 3 were robotic (21%). Planned surgeries included liver resection (*n* = 6, 43%), cholecystectomy (*n* = 4, 29%), pancreatic resection (*n* = 2, 14%), and staging laparoscopy (*n* = 2, 14%) (Table 1).

**Table 1:** Characteristics of 14 patients who underwent FlagNavi and conventional IOUS

Sex, female	7 (50%)	(50%)
Age	68	(41–92)
Approach		
Laparoscopic ≥	11	(79%)
Robotic	3	(21%)
Planned surgery		
Liver resection	6	(43%)
Cholecystectomy	4	(29%)
Pancreatic resection	2	(14%)
Staging laparoscopy	2	(14%)

### Target identification

Twenty-six anatomical targets were investigated using both Flag AR and conventional IOUS. To minimize the influence of surgeons’ memory of target locations, approximately half of the targets were examined with Flag AR first, while the remaining half were examined with conventional IOUS first. These included liver tumors (*n* = 13), gallbladder tumors (*n* = 4), cystic artery (*n* = 3), confluence of hepatic vein (*n* = 3), pancreatic tumors (*n* = 2), and centro-inferior portal vein (*n* = 1) (Table 2).

**Table 2:** Details of investigated targets (*n* = 26)

Liver tumor	13
Gallbladder tumor	4
Cystic artery	3
Confluence of hepatic vein (V8 to MHV)	3
Pancreatic tumor	2
Centro-inferior portal vein	1

### Gaze tracking analysis

**Fig. 3:**
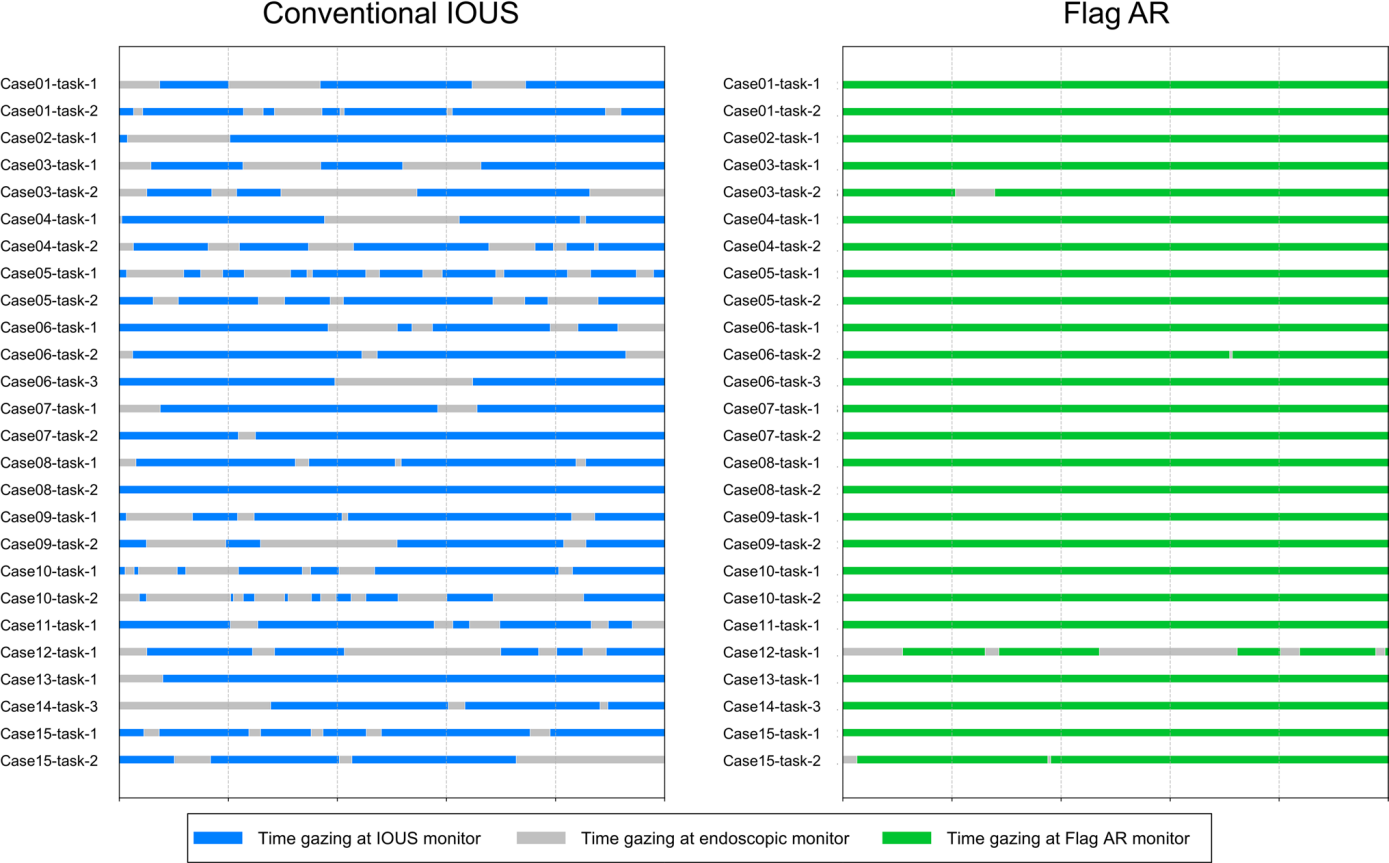
Timeline analysis of surgeon gaze distribution during target identification tasks. Timeline visualization of surgeon visual attention patterns during intraoperative ultrasonography (IOUS) target identification across multiple cases. Left panel (Conventional IOUS): Surgeons exhibit frequent alternating gaze shifts between the endoscopic monitor (blue) and separate IOUS display (gray), demonstrating the divided attention pattern characteristic of dual-monitor workflows. Right panel (Flag AR): Surgeons demonstrate predominantly continuous focus on the endoscopic monitor with Flag AR overlay (green) with minimal attention shifts to other displays (gray), indicating sustained single-display concentration throughout the task duration

Flag AR significantly reduced gaze shifts compared to conventional IOUS (1.8 vs 8.4 shifts, *P* < 0.001). Figure 3 shows timeline analysis of surgeon gaze distribution on two monitors during target identification tasks. Time focused on a single monitor was significantly higher with Flag AR (97.9% vs. 73.8%, *P* < 0.001). Average task completion time showed no significant difference between modalities (23.8 vs. 22.1 s, *P* = 0.351) (Table 3).

**Table 3:** Comparison of surgeon gaze patterns and target identification performance between flag AR and conventional IOUS

	With flag AR	Conventional IOUS	*P* value
Number of gaze shifts	1.8	8.4	< 0.001
Time focused on a single monitor (%)	97.9	73.8	< 0.001
Average task completion time (second)	23.8	22.1	0.351

## Discussion

This study presents the first clinical validation of Flag AR, an AR-based IOUS superimpose system that improves surgeon visual workflow efficiency during minimally invasive hepatobiliary–pancreatic surgery. Our findings demonstrate that surgeons using Flag AR could focus on a single endoscopic monitor for over 97% of the time when searching for targets with IOUS. This improvement can fundamentally transform the spatial visualization of endoscopic surgery.

Flag AR demonstrates an approach in intraoperative imaging technology by achieving precise AR superimposition using exclusively visual markers, eliminating the need for complex magnetic field generators or bulky additional positioning sensors commonly required in existing imaging systems [13–16]. To our knowledge, this is the first clinical series demonstrating endoscopic ultrasound overlay technology using purely visual marker-based tracking in minimally invasive surgery [17, 18]. This approach offers several advantages: reduced setup complexity [19], elimination of electromagnetic interference concerns [3], and enhanced portability that makes the system readily adaptable to various surgical environments.

A key clinical benefit of Flag AR lies in its ability to improve surgeons’ understanding of the spatial relationship between visible organ surfaces on the endoscopic monitor and embedded anatomical structures. During MIS procedures, tumors and vascular structures embedded in solid organs such as the liver and pancreas remain invisible even on magnified endoscopic views. Flag AR addresses this limitation by creating a direct visual connection between the endoscopic field and the underlying anatomy through ultrasonography, allowing surgeons to perceive embedded structures in spatial context with the organ surface. While quantitative evaluation for spatial recognition was not performed in this study, our findings support the enhancement of spatial comprehension by Flag AR. The surgeons’ sustained visual focus on a single display, which occurred in 97.9% of the time during target searches using IOUS, may be indicative of reduced confusion and enhanced spatial understanding. This sustained attention pattern suggests that the surgeons were not experiencing disorientation or difficulty in target interpretation.

Several limitations should be acknowledged in the present study. First, the evaluation was conducted in a relatively small cohort with a limited number of complex procedures such as hepatectomy for deeply located liver tumors or multinodular lesions. Larger studies including more challenging cases are needed to fully evaluate the clinical impact of Flag AR technology. Second, the current Flag AR system superimposes grayscale IOUS images onto color endoscopic displays, requiring surgeons to have adequate familiarity with ultrasound interpretation. Future developments incorporating color Doppler or contrast enhancement may improve the interpretability of superimposed IOUS images. Third, the Flag AR IOUS probe design has inherent mechanical limitations due to port-based insertion and flag-shaped configuration. In this study, one target (a liver tumor located in the deep right lateral sector) was inaccessible due to these constraints. Future hardware developments, including robotic arm-assisted manipulation, may expand accessibility. Next, research should focus on conducting larger multicenter studies to validate the clinical benefits of Flag AR technology across diverse patient populations and surgical procedures. Integration of advanced ultrasound modalities such as contrast-enhanced ultrasound or elastography may further enhance the system’s diagnostic capabilities. Additionally, the development of artificial intelligence algorithms for automated target recognition and surgical guidance represents an exciting frontier for AR-based surgical navigation systems.

In conclusion, the present study demonstrates that Flag AR can improve surgeon visual workflow efficiency during various minimally invasive hepatobiliary–pancreatic surgeries. This AR-based IOUS superimposition system represents the first implementation of purely visual marker-based endoscopic ultrasound overlay technology, with potential for enhancing spatial visualization and reducing cognitive workload during complex surgical procedures in MIS.

## Supplementary Information

Below is the link to the electronic supplementary material.

Supplementary file1 (MP4 187047 KB)
